# Human polyomavirus JC replication and non-coding control region analysis in multiple sclerosis patients under natalizumab treatment

**DOI:** 10.1007/s13365-015-0338-y

**Published:** 2015-05-01

**Authors:** Valeria Pietropaolo, Anna Bellizzi, Elena Anzivino, Marco Iannetta, Maria Antonella Zingaropoli, Donatella Maria Rodio, Manuela Morreale, Simona Pontecorvo, Ada Francia, Vincenzo Vullo, Anna Teresa Palamara, Maria Rosa Ciardi

**Affiliations:** Department of Public Health and Infectious Diseases, Sapienza University of Rome, P.le Aldo Moro, 5, 00185 Rome, Italy; Sbarro Institute for Cancer Research and Molecular Medicine, Center for Biotechnology, College of Science and Technology, Temple University, Philadelphia, USA; Department of Public Health and Infectious Diseases, Institute Pasteur, Cenci-Bolognetti Foundation, Sapienza University of Rome, Rome, Italy; Department of Neuroscience, Temple University School of Medicine, Philadelphia, USA; Institut Cochin, Inserm U1016, Equipe Physiologie des cellules dendritiques, Paris, France; Department of Medico-Surgical Sciences and Biotechnologies, Section of Neurology, Sapienza University of Rome, Rome, Italy; Istituto di Ricerca e Cura a Carattere Scientifico—Neuromed, Pozzilli, Isernia Italy; Multiple Sclerosis Center, Department of Neurology and Psychiatry, Sapienza University of Rome, Rome, Italy; San Raffaele Pisana Scientific Institute for Research, Hospitalization and Health Care, Rome, Italy

**Keywords:** Polyomavirus JC, Multiple sclerosis, Natalizumab, STRATIFY JCV^®^, Real-time PCR, NCCR sequencing

## Abstract

In the last years, the treatment of multiple sclerosis (MS) patients with natalizumab has been associated with the occurrence of progressive multifocal leukoencephalopathy (PML) caused by human polyomavirus JC (JCV). Here, we have shown a significant correlation between patients with JC viruria and positive JC-specific antibody response and patients without JCV-specific antibodies after 1 year of natalizumab (*p* = 0.0006). Furthermore, JCV-specific quantitative PCR on urine and plasma samples, collected at the enrollment (t0) and every 4 months (t1, t2, t3) in the first year and at two time points (t4 and t5) in the second year of natalizumab treatment, indicated the prevalence of JC viremia rather than JC viruria only in the second year of treatment (*p* = 0.04). Moreover, the analysis of JCV non-coding control region (NCCR) sequences in peripheral blood mononuclear cells of patients with JC-specific antibodies after 12 natalizumab infusions (t3) revealed the presence of rearranged sequences, whereas the prevalence of genotypes 1A, 1B, and 4 was detected in these patients by VP1 sequence analysis. In summary, JC viruria evaluation seems to be useful to identify early those patients who do not already develop a humoral immune response against JCV. It may also be interesting to study the JCV NCCR rearrangements since they could give us new insights on the onset of neuro-invasive viral variants.

## Introduction

In the last years, many immune-mediated diseases such as multiple sclerosis (MS) have been managed using biological drugs. Nevertheless, the use of these drugs was soon associated with the onset of progressive multifocal leukoencephalopathy (PML). In 2005, two patients with MS (Kleinschmidt-DeMasters and Tyler 2005; Langer-Gould et al. 2005) and one with Crohn’s disease (CD) (Van Assche et al. 2005) were reported as the first cases of PML development after the treatment with natalizumab (Tysabri^®^, Biogen Idec, Elan Pharmaceuticals). PML is a demyelinating disease of the central nervous system (CNS) caused by polyomavirus JC (JCV) lytic infection of oligodendrocytes. JCV is a DNA neurotropic virus isolated in 1971 from the brain of a patient with PML (Padgett et al. 1971). Ninety percent of the worldwide adult population has antibodies against JCV, with 27 % of healthy individuals that asymptomatically release virions in the urine (Brew et al. 2010; Kean et al. 2009). The early and the late coding regions of JCV genome are highly conserved and can be associated with viral genotypes and subtypes of specific geographical areas (Agostini et al. 2001). In contrast, the viral non-coding control region (NCCR) is highly variable and characterized by determinants of neurotropism and neurovirulence. Rearranged NCCR sequences were observed during the host immunosuppression, and they correlated with a poor prognosis in PML patients. The incidence of PML has increased during the global HIV epidemic in the 1980s (Tan and Koralnik 2010), although the introduction of combination antiretroviral therapy (cART) in the treatment of HIV infection has overturned this trend, with a reduction of PML onset among HIV patients. However, in recent years, PML cases are increasing among patients with immune-mediated diseases treated with monoclonal antibodies (mAb) (Weissert 2011), and as of January 2015, 514 cases of PML had been reported among the 132,600 MS patients treated with natalizumab worldwide, with a fatality rate of about 20–25 % (TYSABRI Safety Update 2015). The estimated risk of natalizumab PML-associated is 11.1 cases per 1000 patients (95 % CI 8.3 to 14.5) when the three main risk factors are present: (1) JCV-specific antibodies, (2) previous use of immunosuppressants, and (3) 25 months of natalizumab treatment (Bloomgren et al. 2012; Sørensen et al. 2012). Natalizumab interferes with lymphocyte trafficking through the blood–brain barrier (BBB) by blocking the interaction of very late antigen 4 (VLA4) with adhesion molecule type 1 on vascular cells (VCAM-1). This blocking seems to result in decreased immunosurveillance and JCV reactivation from latency (Berger and Houff 2009; Tan et al. 2010; Zohren et al. 2008). To date, specific biomarkers of PML risk assessment are not available and no correlation has been reported between the JC viral load in blood and/or urine and the PML onset (Andreoletti et al. 2002; Marzocchetti et al. 2009; Rudick et al. 2010; Tan et al. 2010). However, a new enzyme immunoassay (STRATIFY JCV^®^) has been distributed by the same pharmaceutical company producing natalizumab to discriminate JCV-specific serostatus (Gorelik et al. 2010) and viral DNA detection in urine samples has been found useful for a more accurate stratification of PML risk in patients treated with natalizumab (Laroni et al. 2012). In fact, it has been suggested that JCV viruria could allow identification of JCV-positive patients with undetectable JCV-specific antibodies (Mancuso et al. 2012). Nevertheless, the exact mechanism by which natalizumab promotes the PML pathogenesis has not yet been well defined, but it strongly depends on a reduced immune surveillance of the central nervous system (Coisne et al. 2009; Kivisakk et al. 2009; Stuve et al. 2006). Despite the JCV lytic infection of oligodendrocytes in individuals with PML, JCV can latently persist in a variety of cell types including hematopoietic precursor CD34+ cells and B cells in the bone marrow, brain, tonsils, and the bloodstream (Marzocchetti et al. 2009; Tan et al. 2010). It is also important to note that the development of PML in immunocompromised patients appears to be limited to rearrangements of the viral NCCR and the consequent enhancing of viral gene expression mediated by the binding of specific cellular transcription factors on the JCV NCCR (Marshall and Major 2010). Therefore, in this study, we have investigated the trend of JCV replication in cohorts of relapsing–remitting MS (RRMS) patients at a different stage of natalizumab treatment: at the baseline with the number of natalizumab infusions equal to 0 (t0), during the first 12 infusions (<12 months), and after more than 12 months of treatment (>12 months). Three aspects of JCV replication have been evaluated: (1) the monitoring of JCV viral load by real-time quantitative PCR (Q-PCR) in different biological samples collected from the cohort at specific sampling times; (2) the analysis of possible rearrangements of the JCV NCCR, in order to identify possible mutations in the binding sites for specific transcription factors; and (3) the sequence analysis of the JCV VP1 gene, in order to establish a possible correlation between a specific JCV genotype/subtype and patients with RRMS treated with natalizumab.

## Materials and methods

### Features of enrolled patients and study schedule

Patients affected by RRMS, referred to the Department of Neurology and Psychiatry of the University of Rome “Sapienza” from March 2012 to March 2014, and with the following number of infusions of natalizumab, have been enrolled:

- Twenty-two patients (mean age ± stand. dev. 30.6 ± 6.8; mean months of illness ± stand. dev. 84 ± 85.7; mean Expanded Disability Status Scale (EDSS) ± stand. dev. 1.9 ± 1.3) were enrolled before treatment with natalizumab (t0: baseline or 0 infusions) and a total of 22 samples of whole blood in EDTA and 22 urine samples have been collected. Of these 22 patients, 2 had discontinued therapy for allergic reaction to the biologic after 2 natalizumab infusions and 5 showed poor compliance (Table 1). The remaining 15 patients (mean age ± stand. dev. 33 ± 7.7; mean months of illness ± stand. dev. 112.4 ± 100.3; mean EDSS ± stand. dev. 1.9 ± 1.3) have been monitored during the first year of treatment with natalizumab (follow-up <12 months) as follows: 1 sample of whole blood in EDTA and 1 tube of urine have been collected after 4 (t1), 8 (t2), and 12 (t3) infusions of natalizumab, respectively. Forty-five samples of whole blood in EDTA and 45 urine samples have been tested (Table 1).

**Table 1:** Clinical features of RRSM patients

	Baseline (0 infusion) t0	<12 months	>12 months	
		4–12 infusions (t1–t3)	13–24 infusions (t4)	>24 infusions (t5)
F/M (*n*)	8/14	13/17	17/5	9/2
Total	22	30	22	11
STRATIFY JCV^®a^ +/− t0	4/18	5/25		
STRATIFY JCV^®a^ +/− t3	–	13/17	6/16	3/8
Mean age (±stand. dev.)	30.6 (±6.8)	35 (±8.4)	38.2 (±7.6)	39.7 (±10.6)
Mean of months of disease (±stand. dev.)	84 (±85.7)	107 (±89.7)	100 (±79.2)	75.6 (±44.9)
Mean EDSS^b^ (±stand. dev.)	1.9 (±1.3)	2.1 (±1.0)	2.2 (±1.1) 2 (1–4)	2.2 (±1.0) 2 (1–4)
No therapy^c^ (*n*)	9	9	5	1
Interferon^c^ (*n*)	13	18	12	8
Glatimer acetate^c^ (*n*)	–	3	5	2

*F* female, *M* male, *n* number of patients, *stand. dev*. standard deviation

^a^STRATIFY JCV^®^: two-step virus-like particle-based enzyme-linked immunosorbent assay (ELISA) was performed at t0 and t3 (1 year of treatment), to detect specific anti-JC virus antibodies in serum of the enrolled subjects (Gorelik et al. 2010)

^b^EDSS: Kurtzke Expanded Disability Status Scale, with values ranging from 0 (normal neurological examination) to 10 (bedridden patient) (Kurtzke 1983)

^c^Therapy before starting with natalizumab treatment
- In addition, 18 whole blood samples in EDTA (4 collected at t1, 4 at t2, and 10 at t3) and 14 urine samples (3 collected at t1, 3 at t2, and 8 at t3) have been collected from other 15 patients with RRMS with the number of infusions ranging from 4 to 12 (<12 months) (Table 1).
- Finally, 38 samples of whole blood in EDTA and 34 urine samples were collected from 22 patients with RRMS (mean age ± stand. dev. 38.2 ± 7.6; mean months of illness ± stand. dev. 100 ± 79.2; mean EDSS ± stand. dev. 2.2 ± 1.1) with the number of infusions ranging from 13 to 24 (t4). Moreover, 18 samples of whole blood in EDTA and 14 urine samples have been collected from 11 patients with RRMS (mean age ± stand. dev. 39.7 ± 10.6; mean months of illness ± stand. dev. 75.6 ± 44.9; EDSS mean ± stand. dev. 2.2 ± 1.0) with the number of infusions higher than 24 (t5) (>12 months) (Table 1).

All patients satisfied the Italian Agency of Drug criteria for natalizumab treatment (AIFA 2010). The therapeutic program consisted of 300 mg intravenous (IV), infused over 1 h, every month.

After the activation of the STRATIFY JCV^®^ service, supported by Biogen Idec, the assessment of specific anti-JC virus antibodies in serum of the enrolled subjects was performed at t0 and t3 (Gorelik et al. 2010).

At each follow-up visit, disease activity has been assessed by the Kurtzke Expanded Disability Status Scale (EDSS), with values ranging from 0 (normal neurological examination) to 10 (bedridden patient). EDSS is a standardized composite neurological examination, developed for multiple sclerosis clinical research (Kurtzke 1983). The magnetic resonance imaging (MRI) has been performed at the beginning of therapy and eventually during treatment, depending on the clinical course of each patient. Finally, the lumbar puncture has been performed only in the case of neuro-radiological or clinical alterations in the presence of JCV replication in plasma or peripheral blood mononuclear cells (PBMCs). Each patient, properly informed, has signed informed consent, in order to take part in the study.

### DNA extraction

DNA has been extracted from a total of 141 plasma, 129 urine, and 141 PBMC samples collected at the specified time-points. DNA extraction has been conducted on 500 μL of urine sample using the DNeasy^®^ Blood & Tissue Kit (QIAGEN, S.p.A, Italy). Blood samples have been collected in 4-mL Vacuntainer^®^ tubes containing EDTA (BD Becton Dickinson, S.p.A, Italy) and centrifuged at 3500 rpm for 10 min and DNA was extracted from 200 μL of plasma by the DNeasy^®^ Blood & Tissue Kit (QIAGEN, S.p.A, Milan, Italy). Peripheral blood mononuclear cells (PBMCs) were isolated from whole blood using a standard Ficoll Hypaque density gradient centrifugation (Bøyum et al. 1991). The viable leukocytes have been determined by trypan blue exclusion, and PBMC DNA extraction has been obtained on 10^6^ cells by the QIAmp^®^ DNA Blood Kit (QIAGEN, S.p.A, Milan, Italy). All extracted DNA samples have been stored at −20 °C until use.

### JCV T-Ag real-time PCR (Q-PCR)

Q-PCR has been performed to detect and quantify the JCV genome copy numbers using a 7300 Real-Time PCR System (Applied Biosystems, USA) and following published protocol (Delbue et al. 2008). This Q-PCR was specific for a 54-bp amplicon in the JCV T antigen region. The viral load results have been reported as the mean of two positive reactions for each sample, and the JCV DNA load in urine and plasma specimens has been expressed as genome equivalents (gEq)/milliliter of sample and as genome equivalents (gEq)/10^6^ cells of sample for PBMCs. In each Q-PCR section, negative and positive controls have been considered. The positive control consisted of serial dilutions (range 10^5^–10^2^ gEq/mL) of a plasmid containing the entire JCV genome, used to fit a standard curve. Finally, the amount of DNA in PBMC samples has been normalized by a specific TaqMan PCR for the housekeeping gene glyceraldehyde-3-phosphate-dehydrogenase (GAPDH, accession no. J04038). Results have been considered acceptable only in the presence of GAPDH positivity (Costa et al. 2009).

### JCV NCCR PCR

Two pairs of primers that anneal to invariant regions flanking the NCCR of JCV (Pietropaolo et al. 2003) have been used in a nested-PCR. BKTT1 (5′-AAG GTC CAT GAG CTC CAT GGA TTC TTC C-3′) and BKTT2 (5′-CTA GGT CCC CCA AAA GTG CTA GAG CAG C-3′) amplified a 724-bp DNA fragment in JCV Mad-1 (Flaegstad et al. 1991). JC1 (5′-CCT CCA CGC CCT TAC TAC TTC TGA G-3′) and JC2 (5′-AGC CTG GTG ACA AGC CAA AAC AGC TCT-3′) amplified an inner sequence of the first PCR product, generating a fragment of 308 bp (Markowitz et al. 1993). PCR products have been analyzed on 2 % agarose gels by ethidium bromide staining.

### JCV VP1 PCR

A 215-bp sequence has been amplified from the viral protein 1 (VP1) gene using primers JLP-15 (5′-ACA GTG TGG CCA GAA TTC ACT ACC-3′) and JLP-16 (5′-TAA AGC CTC CCC CCC AAC AGA AA-3′) (Agostini et al. 2001). After denaturation at 95 °C for 9 min, 40 cycles at 95 °C for 40 s, 63 °C for 40 s, and 72 °C for 40 s have been concluded with a final extension at 72 °C for 7 min. PCR products have been analyzed using 2 % electrophoresis agarose gel stained by ethidium bromide (Monaco et al. 2001).

### Sequencing of JCV NCCR and VP1 regions

PCR products corresponding to JCV NCCR and VP1 regions have been purified with the QIAquick PCR purification kit, according to QIAGEN protocol (Pietropaolo et al. 2003). DNA sequencing has been conducted *in service* (BioFab research s.r.l., Rome, Italy). All NCCR sequences were compared to the prototype Mad-1 (NCBI Reference Sequence: NC_001699.1) (Frisque et al. 1984) and archetype CY (Yogo et al. 1990), whereas the sequences obtained from amplification of the VP1 region were analyzed on the basis of single nucleotide polymorphisms (SNPs) used to classify the JCV genotypes/subtypes (Jobes et al. 2001). Sequence alignments were performed with ClustalW2 at the EMBL-EBI website using default parameters (ClustalW2 2014).

### Data analysis

Data were shown as mean ± standard deviation or medians and ranges. If the *Z* test indicated a non-normal distribution, non-parametric tests such as Mann–Whitney *U* test and Kruskal–Wallis test have been used. Categorical data were analyzed by *χ*^2^ test and Student’s *t* test. *p* values <0.05 were considered significant.

## Results

### Evaluation of JCV-specific serum antibodies by STRATIFY JCV^®^ and JCV load by Q-PCR in biological samples collected at t0 from 22 patients with RRMS

Twenty-two samples of whole blood in EDTA and 22 samples of urine were collected, and JCV-specific antibodies were observed in serum of 4 patients (STRATIFY JCV^®^ positive) while the other 18 patients were tested STRATIFY JCV^®^ negative. Among the 4 STRATIFY^®^ JCV-positive patients, viral DNA was detected exclusively in plasma (2.84 log10 gEq/mL) and in PBMCs (2.07 log10 gEq/10^6^ cells) of 1 patient (Table 2). By contrast, in 18 STRATIFY JCV^®^-negative patients, JC viruria was found in 4/18 samples with a median viral load of 4.38 log10 gEq/mL (range 3.48–4.58), while JC viremia was observed in 2/18 patients with a median viral load of 3.02 log10 gEq/mL (range 2.70–3.20). Moreover, in these 18 patients, JCV DNA was detected in 2 samples of PBMCs with a median viral load of 3.42 log10 gEq/10^6^ cells (range 1.95–3.72) (Table 2). At t0, no statistically significant difference and correlation were observed between viruria and/or viremia and STRATIFY^®^ JCV results in these patients.

**Table 2:** JCV load and STRATIFY JCV^®^ of RRSM patients at baseline (t0)

Pt (*n*)	Pt JCV DNA+ (*n*)^b^	Pt JCV DNA− (*n*)^b^	Urine		Plasma		PBMC	
			JCV+/JCV−	log10 gEq/mL (range)^c^	JCV+/JCV−	log10 gEq/mL (range)^c^	JCV+/JCV−	log10 gEq/10^6^ c (range)^c^
STRATIFY JCV^®a^ positive								
4	1	3	0/4	–	1/3	2.84	1/3	2.07
STRATIFY JCV^®a^ negative								
18	7	11	4/14	4.38 (3.48–4.58)	2/16	3.02 (2.70–3.20)	2/16	3.42 (1.95–3.72)
Total (*n*)	8/22	14/22	4/18	4.38 (3.48–4.58)	3/19	2.84 (2.70–3.20)	3/19	2.07 (1.95–3.72)

*n* number of patients, *Pt* patients

^a^STRATIFY JCV^®^: two-step virus-like particle-based enzyme-linked immunosorbent assay (ELISA) was performed at t0, to detect specific anti-JC virus antibodies in serum

^b^Pt JCV DNA+ and Pt JCV DNA−: number of patients with or without JCV DNA in at least 1 sample of plasma and/or PBMCs and/or urine

^c^JCV load values were expressed as median (range) of log10 genome equivalent (gEq)/mL in urine and in plasma, and as median (range) log10 gEq/10^6^ cells in PBMCs (gEq/10^6^ c)

### Evaluation of JC viral load by Q-PCR in biological samples of 15 RRMS patients with follow-up in the first year of treatment with natalizumab (follow-up <12 months)

**Fig. 1:**
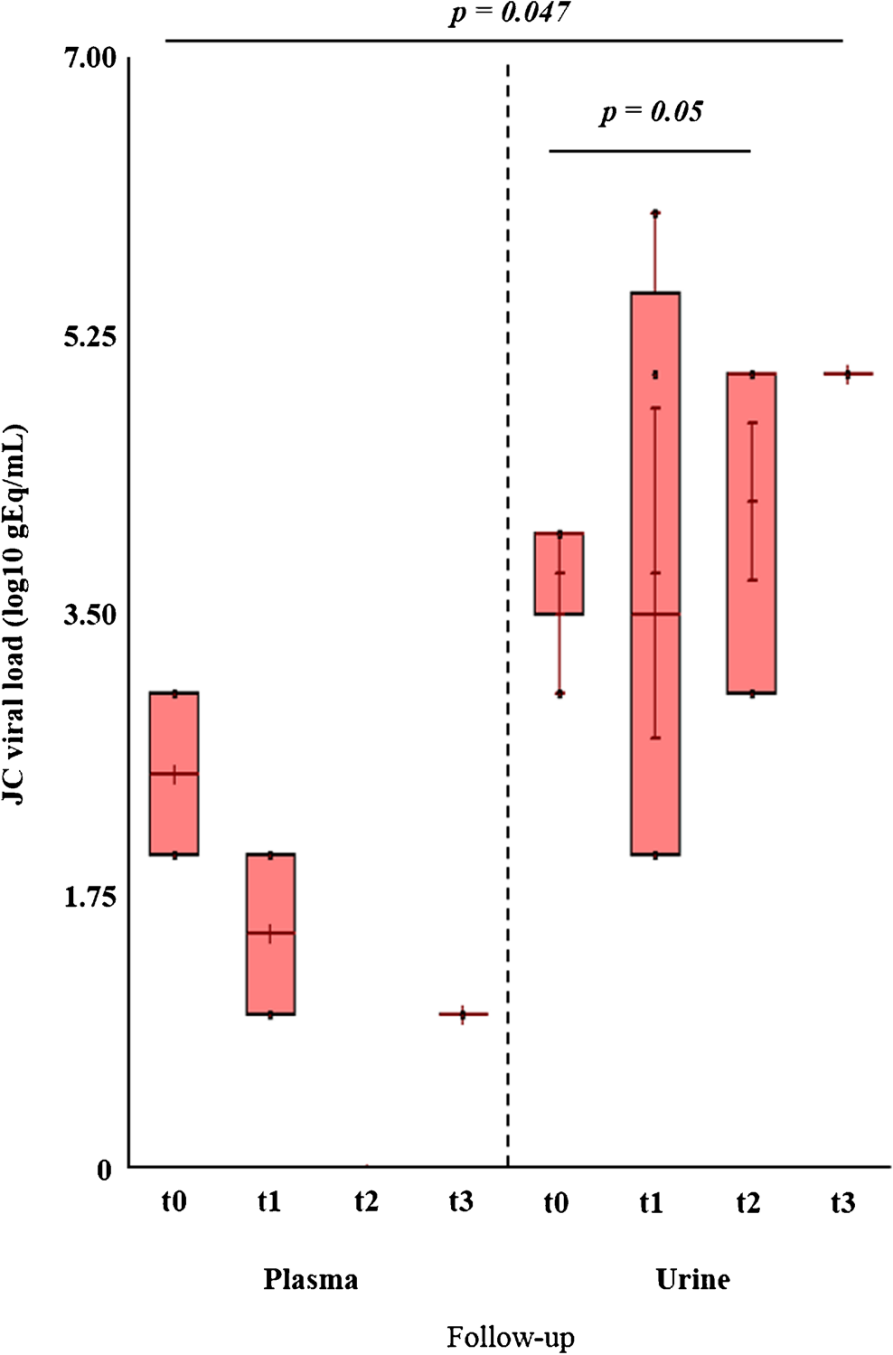
JC viremia and viruria in 15 RRSM patients during the first 12 months of natalizumab treatment (follow-up <12 months). Serial urine and plasma samples were performed at t0 (baseline: 0 natalizumab infusions) and every 4 months for 1 year (t1: 4 infusions; t2: 8 infusions; t3: 12 infusions). There was no statistically significant difference between JC viremia values recorded at t0 and the other values observed at each time of follow-up. On the other hand, JC viruria values observed at t2 were significantly higher than those observed at t0. Finally, at each time of follow-up, the JC viremia values were always significantly lower with respect to the JC viruria values. Statistical analysis was performed using nonparametric tests and significance was set for a *p* value <0.05

At t0, JCV-specific antibodies were detected only in 1/15 patient, while the number of STRATIFY JCV^®^-positive patients rose to 7/15 at t3. Regarding the detection of JCV DNA by Q-PCR in urine, in samples collected at t0, JC viruria was observed in 4/15 STRATIFY JCV^®^-negative patients at t0. These patients developed anti-JCV antibodies during the first year of treatment with natalizumab, becoming STRATIFY JCV^®^ positive at t3. The median viral load in urine samples at t0 was 4.38 log10 gEq/mL (range 3.48–4.58), while after 4 months of treatment with natalizumab (t1), this value was 4.11 log10 gEq/mL (range 2.00–6.01). At t2 (after 8 natalizumab infusions), the number of patients with JCV DNA in the urine increased from 4 to 5, with the finding of JC viruria in 1 patient which resulted STRATIFY JCV^®^ negative both at t0 and at t3. This patient subsequently became negative for JCV DNA in urine at t3 (after 12 natalizumab infusions). In conclusion, a persistent viruria throughout follow-up was observed in 4/15 RRMS patients. Overall, compared to t0, the median viral load in the urine increased up to 5.18 log10 gEq/mL (range 3.77–5.65) at t2 and up to 5.63 log10 gEq/mL (range 5.29–5.94) at t3, and this increase was statistically significant (*p* = 0.05) (Fig. 1).

Regarding the JCV DNA detection in plasma samples, JC viremia was found at t0 in only 2 patients with a negative STRATIFY JCV^®^ and a value of the median viral load of 2.95 log10 gEq/mL (range 2.70–3.21). However, these 2 patients with viremia at t0 were negative at t1, while other 2 patients (STRATIFY JCV^®^ negative at t0 and STRATIFY JCV^®^ positive at t3) with persistent viruria throughout the follow-up showed JCV DNA in plasma with a median viral load of 2.15 log10 gEq/mL (range 1.61–2.69). Finally at t2, all 15 RRMS patients were negative for JCV DNA in plasma whereas, at t3, only 1 patient (STRATIFY JCV^®^ negative both at t0 and t3) showed JCV load in plasma (1.86 log10 gEq/mL) and in PBMCs (1.11 log10 gEq/10^6^ cells) (Fig. 1).

Regarding the PBMCs, at t0, JCV DNA was detected in PBMCs of 2 patients, STRATIFY JCV^®^ negative at t0 and STRATIFY JCV^®^ positive t3, with a mean value of viral DNA copies of 2.82 log10 gEq/10^6^ cells (range 1.92–3.72). One of these two patients showed persistent viruria throughout the follow-up. At t1, the viral DNA was found in the PBMCs of only 1 patient (1 log10 gEq/10^6^ cells) with viremia at the same time of sampling and persistent viruria throughout the follow-up. Finally, at t2, in only 1 patient was JCV DNA detected in PBMCs, with a viral load of 2.16 log10 gEq/10^6^ cells (data not shown).

Statistical analysis of these data highlighted that the median number of genome equivalents of JCV detected in urine was significantly higher than that observed in plasma (*p* = 0.047) (Fig. 1) and in PBMC (*p* = 0.029) (data not shown). Finally, 2/15 patients did not perform two natalizumab infusions, due to a concomitant urinary infection by *Escherichia coli* and a clinically worsening of RRMS with a positive STRATIFY JCV^®^ at t3.

### Evaluation of JC viral load by Q-PCR in biological samples of 30 RRMS patients with the number of natalizumab infusions ranging from 4 to 12 (<12 months)

In addition to the 15 patients described above, in this cohort were also enrolled another 15 patients with the mean number of infusions between 4 and 12 (<12 months). From these 15 patients, 18 whole blood samples in EDTA (4 obtained at t1, 4 at t2, and 10 at t3) and 14 urine samples (3 obtained at t1, 3 at t2, and 8 at t3) were collected and the JC viral load was assessed by q-PCR. Regarding the blood samples, the results showed that all 18 plasma samples were negative for viral DNA, whereas only 1/18 PBMC sample, obtained from a STRATIFY JCV^®^-positive patient at t3, showed a JC viral load of 1.95 log10 gEq/10^6^ cells. On the other hand, concerning the 14 urine samples, in only one sample from 1 STRATIFY JCV^®^-negative patient at t3 was JC viruria observed with a viral load value of 6.04 log10 gEq/10^6^ cells.

Among the 59 urine samples collected in this cohort, 45 belonged to the 15 RRMS patients with a complete follow-up in the first year of natalizumab treatment and 14 belonged to 15 RRMS patients with the number of natalizumab infusions ranging from 4 to 12 (<12 months). The results obtained by Q-PCR showed that 14/59 urine samples were positive to the JCV DNA and 12 of these 14 positive urine samples were collected from 4 patients with a negative STRATIFY JCV^®^ at t0 and a positive STRATIFY JCV^®^ at t3. The median value of these 12 samples with JC viruria was 5.33 log10 gEq/mL (range 2–6.01). In the remaining 2 urine samples, taken from two patients with a negative STRATIFY JCV^®^ both at t0 and t3, the mean value of the JC viral load was 5.74 log10 gEq/mL (range 3.77–6.04) (Table 3).

**Table 3:** JCV load and STRATIFY JCV^®^ of RRSM patients with the number of natalizumab infusions ranging from 4 to 12 (<12 months)

Pt (*n*)	Pt JCV DNA+ (*n*)^b^	Pt JCV DNA− (*n*)^b^	Urine		Plasma		PBMC		RR (IC 95 %) *p* value^d^
			JCV+ /JCV−	log10 gEq/mL (range)^c^	JCV+ /JCV−	log10 gEq/mL (range)^c^	JCV+/JCV−	log10 gEq/10^6^ c (range)^c^	
STRATIFY JCV^®a^ positive									
13	5	8	12/15	5.33 (2–6.01)	2/27	2.42 (1.61–2.69)	3/26	1.95 (1–2.66)	2.40 (1.52–3.80) *p* = *0.0012*
STRATIFY JCV^®a^ negative									
17	3	14	2/30	5.74 (3.77–6.04)	1/33	1.86	1/33	1.11	
Total (*n*)	8/30	22/30	14/45	5.33 (2–6.04)	3/60	1.86 (1.61– 2.69)	4/59	1.53 (1–2.66)	
RR (IC 95 %)			2.57 (1.61–4.09)						
*p* value^d^			*p* = *0.0006*						

*p* = 0,0012: cases of JCV DNA in urine are statistically associated with a positive STRATIFY JCV® at t3 compared to cases of JCV DNA in plasma

*p* = 0,0006: statistical significant association between number of JCV DNA positive urine samples and positive STRATIFY JCV® at t3 compared to number of JCV DNA positive urine samples and a negative STRATIFY JCV® at t3

*n* number of patients, *Pt* patients

^a^STRATIFY JCV^®^: 2-step virus-like particle-based enzyme-linked immunosorbent assay (ELISA) was performed t3, to detect specific anti-JC virus antibodies in serum

^b^Pt JCV DNA+ and Pt JCV DNA−: number of patients with or without JCV DNA in at least 1 sample of plasma and/or PBMC and/or urine

^c^JCV loads values were expressed as median (range) of log10 genome equivalent (gEq)/mL in urine and in plasma, and as median (range) log10 gEq/10^6^ cells in PBMCs (gEq/10^6^ c)

^d^Relative risk (RR) and 95 % confidence interval (95 % CI) statistically significant with a *p* value <0.05 by *χ* ^2^ test

Regarding the whole blood in EDTA samples, 63 plasma samples and 63 PBMC samples were collected. 3/63 plasma and 4/63 PBMC samples resulted JCV DNA positive. In particular, the mean JC viremia was 2.42 log10 gEq/mL (range 1.61–2.69) in 2 plasma samples collected from 2 patients with a positive STRATIFY JCV^®^ at t3 and a persistent JC viruria during the first year of natalizumab treatment. Moreover, a third patient, with negative STRATIFY JCV^®^ at t0 and t3, showed JCV DNA in both plasma (1.86 log10 gEq/mL) and PBMCs (1.11 log10 gEq/10^6^ cells) (Table 3). Finally, in 3 PBMC samples taken from three patients, with a positive STRATIFY JCV^®^ at t3, a median value of 1.95 log10 gEq/10^6^ cells was quantified (Table 3).

The statistical analysis, performed by the *χ*^2^ test, has allowed verification of a statistically significant association between the number of JCV DNA-positive urine samples and a positive STRATIFY JCV^®^ at t3 with respect to the number of JCV DNA-positive urine samples and a negative STRATIFY JCV^®^ at t3 (*p* = 0.0006). A relative risk (RR) of viral reactivation with urinary shedding equal to 2.57 (95 % CI 1.61–4.09) was also estimated (Table 3). Therefore, the analysis of results allowed us to conclude that the detection of JC viruria by Q-PCR may represent a predictor of JCV reactivation in the first year of natalizumab treatment.

### Evaluation of JC viral load by Q-PCR in biological samples of RRMS patients with the number of natalizumab infusions >12 (>12 months)

In this cohort were enrolled 22 patients with the number of natalizumab infusions ranging from 13 to 24 (t4) and 11 patients with the number of natalizumab infusions >24 (t5) (Table 1). Thirty-eight samples of whole blood in EDTA and 34 urine samples were collected from the 22 patients at t4. Finally, 18 samples of whole blood in EDTA and 14 urine samples were collected from the 11 patients at t5 listed above. In these two groups of patients, there was no statistically significant difference or correlation between the clinical and demographic data, the results of JCV DNA detection in the different biological samples, and the outcomes of STRATIFY JCV^®^. Therefore, all results were related to the cohort of RRMS patients with the number of natalizumab infusions greater than 12. It was important to note that 6 patients (1 patient with a positive STRATIFY JCV^®^ at t3 and 5 patients with a negative STRATIFY JCV^®^ at t3) underwent sample collection at both t4 and t5. Therefore, the total number of patients with more than 13 natalizumab infusions was 27. From these 27 patients, 48 urine, 56 plasma, and 56 PBMC samples were collected. 8/48 urine samples (median JC viruria 5.11 log10 gEq/mL; range 2.94–6.67), 9/56 plasma samples (median JC viremia 3.01 log10 gEq/mL; range 1.52–4.72), and 6/56 PBMC samples (median copies number 2.39 log10 gEq/10^6^ cells; range 1.48–4.11) were positive for JCV DNA These data are summarized in Table 4.

**Table 4:** JC viral load and STRATIFY JCV^®^ in biological samples of RRMS patients with the number of natalizumab infusions >12 (>12 months)

Infusions (*n*)	Pt (*n*)	Pt JCV DNA+ (*n*)^b^	Pt JCV DNA− (*n*)^b^	Urine		Plasma		PBMC	
				JCV+/JCV−	log10 gEq/mL (range)^c^	JCV+/JCV−	log10 gEq/mL (range)^c^	JCV+/JCV−	log10 gEq/10^6^ c (range)^c^
STRATIFY JCV^®a^ positive									
t4	6	3	3	2/5	5.57 (5.50–5.65)	1/8	1.52	1/8	1.66
t5	3	1	2	0/3	–	1/3	4.58	0/4	–
STRATIFY JCV®^a^ negative									
t4	16	7	9	2/25	5.19 (3.81–5.49)	6/23	3.00 (2.67–4.72)	2/27	2.42 (2.23–2.56)
t5	8	4	4	4/7	4.53 (2.94–6.67)	1/13	3.25	3/11	3.03 (1.48–4.11)
Total (*n*)	27^d^	14^d^	13^d^	8/40	5.11 (2.94–6.67)	9/47	3.01 (1.52–4.72)	6/50	2.39 (1.48–4.11)

*n* number of patients, *Pt* patients, *t4* 13–24 natalizumab infusions, *t5* >24 natalizumab infusions

^a^STRATIFY JCV^®^: two-step virus-like particle-based enzyme-linked immunosrobent assay (ELISA) was performed at t3 (after 12 natalizumab infusions), to detect specific anti-JC virus antibodies in serum

^b^Pt JCV DNA+ and Pt JCV DNA−: number of patients with or without JCV DNA in at least 1 sample of plasma and/or PBMC and/or urine

^c^JCV load values were expressed as median (range) of log10 genome equivalent (gEq)/mL in urine and in plasma, and as median (range) log10 gEq/10^6^ cells in PBMCs (gEq/10^6^ c)

^d^For 1 patient with a positive STRATIFY JCV^®^ at t3 and 5 patients with negative STRATIFY JCV^®^ at t3, samples collected both at t4 and t5 were available

### Comparison of JC viremia and viruria in RRMS patients with infusion number equal to 0 (t0: baseline), ranging from 4 to 12 (<12 months) and >12 (>12 months)

**Fig. 2:**
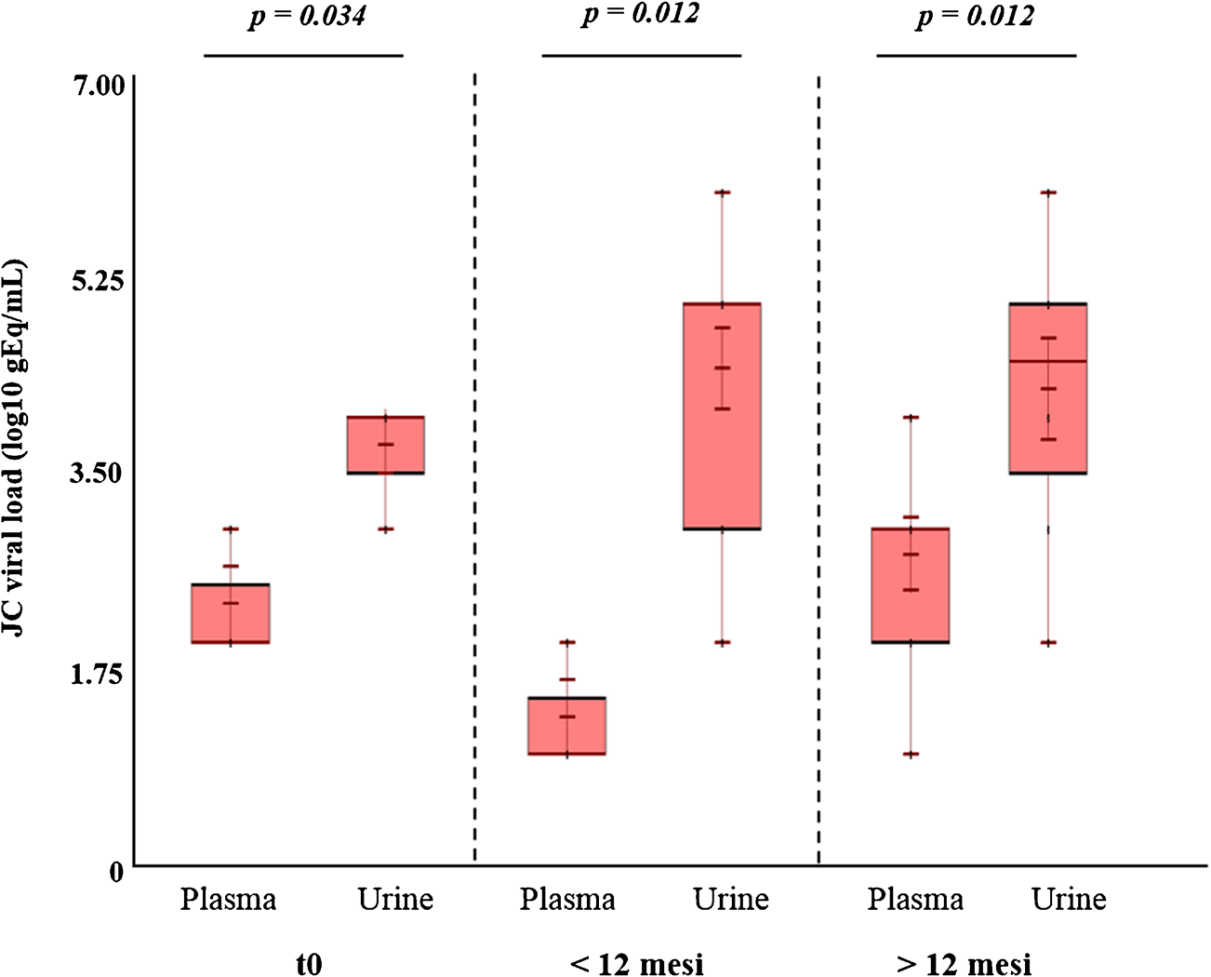
JC viremia and viruria in 22 RRMS patients with the infusion number equal to 0 (t0), 30 RRMS patients treated with natalizumab for less than 12 months (<12 months), and 27 RRMS patients treated for more than 12 months (>12 months). The values of JC viremia were always significantly lower than those of JC viruria in each group of RRMS patients analyzed. All the information related to JCV load values is reported in Table 5. Statistical analysis was performed using non-parametric tests and the significance fixed for a *p* value <0.05

Comparing the values of JC viruria and viremia (log10 gEq/mL) obtained from 22 RRMS patients at t0, 30 RRMS patients treated with natalizumab for less than 12 months, and 27 RRMS patients treated for more than 12 months, it was observed that the median values of JC viremia were always lower than that of JC viruria (*p* = 0.001) (Table 5 and Fig. 2). In addition, it was assessed that patients treated with natalizumab for more than 12 months had a relative risk of 1.71 to develop JC viremia with respect to patients treated with less than 12 months (*p* = 0.04) (Table 5).

**Table 5:** JC viral load in biological samples of RRMS patients with infusion number equal to 0 (t0: baseline), range from 4 to 12 (<12 months) and >12 (>12 months)

Pt (*n*)		Pt JCV DNA+ (*n*)^a^	Pt JCV DNA− (*n*)^a^	Urine		Plasma		PBMC	
				JCV+/JCV−	log10 gEq/mL (range)^b^	JCV+/JCV−	log10 gEq/mL (range)^b^	JCV+/JCV−	log10 gEq/10^6^ c (range)^b^
t0	22	8	14	4/18	4.38 (3.48–4.58)	3/19	2.84 (2.70–3.20)	3/19	2.07 (1.95– 3.72)
<12 months	30	8	22	14/45	5.33 (2–6.04)	3/60	1.86 (1.61–2.69)	4/59	1.53 (1–2.66)
>12 months	27	14	13	8/40	5.11 (2.94–6.67)	9/47	3.01 (1.52–4.72)	6/50	2.39 (1.48– 4.11)
RR						1.71			
(95 % CI)						(1.61–2.52)			
*p* value^c^						*p* = *0.04*			

*p* = 0.04: statistical significant association between number of patients with JC viremia and the treatment with natalizumab for more than 12 months compared to number of patients with JC viremia and the treatment with the same drug for less than 12 months

*n* number of patients, *Pt* patients

^a^Pt JCV DNA+ and Pt JCV DNA−: number of patients with or without JCV DNA in at least 1 sample of plasma and/or PBMC and/or urine

^b^JCV load values were expressed as median (range) of log10 genome equivalent (gEq)/mL in urine and in plasma, and as median (range) log10 gEq/10^6^ cells in PBMCs (gEq/10^6^ c)

^c^Relative risk (RR) and 95 % confidence interval (95 % CI) statistically significant with a *p* value <0.05 by *χ* ^2^ test

### Sequence analysis of JCV NCCR and VP1 in biological samples positive for viral DNA

**Fig. 3:**
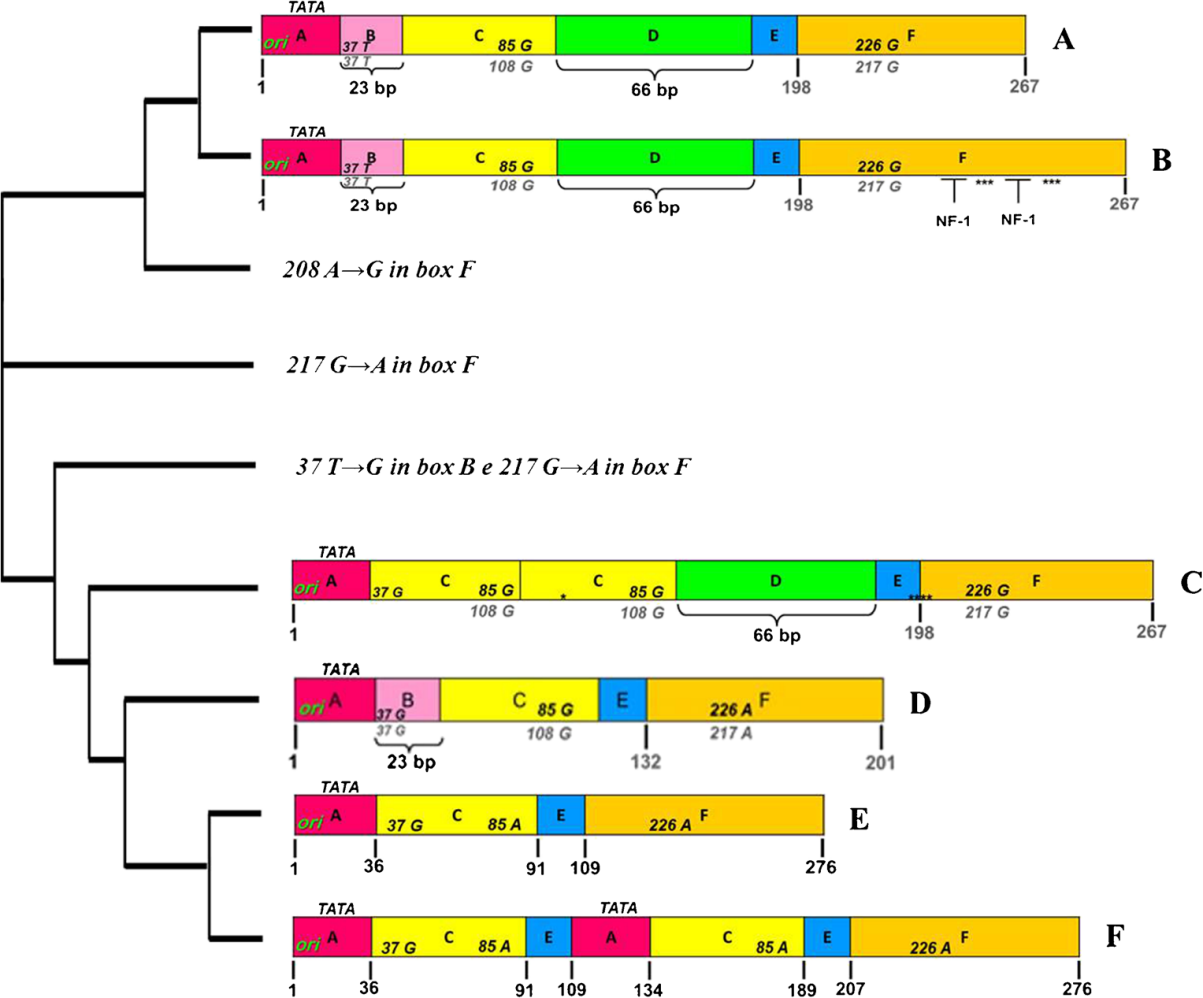
Sequences of JCV NCCR found in biological samples of RRMS patients treated with natalizumab. The nucleotide sequences of JCV NCCR were shown from the core of the replication origin (ori) up to the start codon of the gene leader (agnoprotein) of the late viral genes. In *A*, the nucleotide numbering of the archetype CY sequence, isolated from Yogo and colleagues in 1990, is shown in *bold gray*. In *F*, the nucleotide numbering was based on the NCCR sequence of the PML-associated variant Mad-1, sequenced by Frisque and colleagues in 1984, and the nucleotide number is indicated in *bold black*. In *B*, the NCCR sequence was characterized by a duplication of cellular transcription factor NF-1 binding site in box F, with loss of the 217 G to A (G → A) nucleotide transition in the same box. It was found in 4 JCV DNA-positive urine samples collected at t0, t1, t2, and t3, respectively, from 1 patient with positive STRATIFY JCV^®^. In *C*, a rearranged NCCR was reported, characterized by the deletion of box B with a T to G nucleotide transversion within the cellular transcription factor Spi-B binding site, the duplication of box C, and the presence of box D. It was isolated from PBMC samples of 2 RRMS patients that were STRATIFY JCV^®^ positive after 1 year of treatment with natalizumab (t3). In *D*, the sequence with an archetype CY-like structural organization was reported but with the deletion of box D and two characteristic point mutations: the 37 T to G (T → G) nucleotide transversion within the binding site for the cellular transcription factor Spi-B in box B and the 217 G to A nucleotide transition in box F. Finally, in *E*, the sequence model IS is illustrated, consisting of a single sequence from 98 bp, as reported by Jensen and Major JCV NCCR organization (Jensen and Major 2001). The cladogram was performed by software ClustalW2 (Clustal-W2)

The sequence analysis of JCV NCCR in biological samples positive for viral DNA was carried out by direct sequencing of PCR products. In the group of RRMS patients treated with natalizumab for less than 12 months (<12 months) was found an archetype CY-like structural organization of NCCR, with 217 G to A nucleotide transition in box F, in 68 % of biological samples analyzed, while in the remaining 32 %, NCCR rearranged sequences were identified. In 4 urine samples collected respectively at t0, t1, t2, and t3 from a single RRMS patient with a positive STRATIFY JCV^®^ at t3, a sequence of NCCR, characterized by a duplication of the binding site for the cellular transcription factor NF-1 in box F, was found without the 217 G to A nucleotide transition in the same box. Furthermore, both in plasma and PBMC samples collected from a RRMS patient with negative STRATIFY JCV^®^ at t3, a 208 A to G nucleotide transition in box F of an archetype CY-like structural organization of NCCR, with involvement of the cellular transcription factor NF-1 binding site, was found. Finally, in 4 PBMC samples belonging to 4 different RRMS patients with positive STRATIFY JCV^®^ at t3, the following structural organizations of NCCR were found:

- One sequence with an archetype CY-like structural organization and two characteristic point mutations: the 37 T to G nucleotide transversion within the binding site for the cellular transcription factor Spi-B in box B and the 217 G to A nucleotide transition in box F
- One sequence with an archetype CY-like structural organization but with the deletion of box D and the two point mutations described above
- Two sequences of rearranged NCCR, characterized by the deletion of box B, the 37 T to G nucleotide transversion within the binding site for the cellular transcription factor Spi-B, the duplication of box C, and the presence of box D (Fig. 3)

Finally, in RRMS patients treated with natalizumab for more than 12 months (>12 months), an archetype CY-like structural organization of NCCR, with the 217 G to A nucleotide transition in box F, was found in all biological samples analyzed, with the exception of a sequence that presented an archetype CY-like organization characterized by the 37 T to G nucleotide transversion within the binding site for the cellular transcription factor Spi-B in box B and the 217 G to A nucleotide transition in box F (Fig. 3). This sequence was found in the PBMC sample of a patient with positive STRATIFY JCV^®^ at t3.

Regarding the sequence analysis of JCV VP1, in order to determine the viral genotype/subtype, in RRMS patients treated with natalizumab for less than 12 months (<12 months), genotypes 1A (40 %) and 1B (50 %) were observed. Moreover, in the patient with a positive STRATIFY JCV^®^ and rearranged NCCR in the urine, a JCV co-infection by genotypes 1A and 2C was observed, while only genotype 1A was found in the plasma of the same patient. In the cohort of patients with RRMS treated with natalizumab for more than 12 months (>12 months), genotypes 1A (31 %), 1B (46 %), and 4 (23 %) were always found in the urine and/or plasma positive for JCV DNA.

## Discussion

The pathogenesis of natalizumab-associated PML onset is still poorly understood, and viral and/or host factors are not available in order to detect those natalizumab-treated patients with high risk to develop PML (Mancuso et al. 2012). A clear association between JC viremia and/or viruria and PML has not been also established. In order to stratify the risk of PML onset among RRMS patients treated with natalizumab, a new ELISA assay for the detection of JCV-specific antibodies (JCV STRATIFY^®^) has been designed. Although this method was extremely sensitive, some authors have found a false-negative rate equal to 2.5 % in a cohort of RRMS patients with viral DNA in the urine but no JCV-specific antibodies in serum (Gorelik et al. 2010).

In this study, the data obtained from STRATIFY JCV^®^ were not always correlated with those obtained by Q-PCR: in fact, the presence of viral DNA in urine samples at baseline (t0) preceded the antibody detection in serum samples after 12 natalizumab infusions (negative STRATIFY JCV^®^ at t0 and positive STRATIFY JCV^®^ at t3). These data were in agreement with those of other studies reported in the literature, which showed an asymptomatic JCV reactivation at the level of the urinary tract in patients with RRMS treated with less than 12 natalizumab infusions (Chen et al. 2009; Sadiq et al. 2010). Therefore, as suggested by other authors (Laroni et al. 2012), the analysis of the results allowed us to conclude that in RRMS patients, the trend of JC viruria may represent a predictor of viral reactivation in the first 12 months of treatment with natalizumab also in cases where the STRATIFY JCV^®^ has given a negative result.

Finally, the results obtained suggest that JCV replication in the urinary epithelium, with concomitant presence of JC viruria, seems not to be due to a lack of infection control by the T cells, but rather to a reduction of lymphocyte diapedesis induced by natalizumab. In fact, as already suggested by other authors, the failure of localization of T lymphocytes at the level of the infected renal epithelium seems to abolish the viral antigen presentation by APCs (Chen et al. 2009; Mancuso et al. 2012). Some authors have also observed a decrease in JCV-specific cell-mediated response, after *in vitro* stimulation of PBMC from RRMS patients with a pool of JCV VP1-specific antigen after 6 and 12 natalizumab infusions, respectively. This decrease was found to be more pronounced in JC viremic patients than in non-viremic, and these results led the authors to conclude that natalizumab has a direct negative effect on the production of IFN-γ by JCV-specific T cells after about 1 year of treatment (12 natalizumab infusions). Therefore, the reduction of JCV-specific cellular response seems to favor the reactivation of JCV in the kidney and its subsequent diffusion in the blood (Chen et al. 2009). Moreover, data obtained from this study showed a higher prevalence of negative STRATIFY JCV^®^ patients who develop JCV viremia following the administration of the number of infusions of natalizumab greater than 12. Therefore, the absence of JCV-specific antibodies in these patients led us to assume that humoral immunity may not be able to keep under control the viral replication at the systemic level.

Regarding the study of NCCR sequences, in the group of RRMS patients treated with natalizumab for less than 12 months (<12 months), an archetype CY-like structural organization of NCCR with 217 G to A nucleotide transition in box F was found in 68 % of biological samples analyzed, while in the remaining 32 % analyzed sequences, rearranged NCCR were identified. In 4 urine samples collected respectively at t0, t1, t2, and t3 from 1 RRMS patient with a positive STRATIFY JCV^®^ at t3, a sequence of NCCR, characterized by a duplication of the binding site for the cellular transcription factor NF-1 in box F, was found without the 217 G to A nucleotide transition in the same box. Finally, in 2 PBMC samples, belonging to 2 different RRMS patients with positive STRATIFY JCV^®^ at t3, a rearranged NCCR was found and it was characterized by the deletion of box B, the 37 T to G nucleotide transversion within the binding site for the cellular transcription factor Spi-B, the duplication of box C, and the presence of box D. In particular, the last described sequence showed that rearranged viral strains might reverse into the bloodstream throughout latency in PBMCs. In particular, this rearranged sequence showed a deletion of the B box with 37 T to G nucleotide transversion in the hematopoietic transcription factor Spi-B binding site, duplications of box C, and the CRE element binding site. This rearrangement was similar to the sequence of neurotropic variants found in patients with PML by some authors (Ferenczy et al. 2012; Marzocchetti et al. 2007). In particular, the potential neurovirulence of this rearranged sequence was conferred by the repeat of the CRE element binding site in box C, a specific enhancer of JCV replication in glial cells (Kumar et al. 1996), and by formation of the high-affinity binding site for the transcription factor hematopoietic Spi-B, the expression of which is elevated in hematopoietic cell lines (CD34+ cells and B cells), where the JC virus establishes latency (Lindberg et al. 2008; Major 2010). In addition, as widely described in the literature, it seems that the use of natalizumab may promote the mobilization of CD34+ cells and B cells infected with JCV. Therefore, these cells may be considered carriers of the virus to the CNS (Major 2009; Neumann et al. 2009). In particular, the 37 T to G transversion nucleotide transforms the typical binding site for the cellular factor Spi-B (5′-AAAAGGGAAGGTA-3′) of the non-pathogenic JCV variant (archetype CY) in that characteristic of PML-associated JCV variant Mad-1 (5′-AAAAGGGAAGGGA-3′), and this could result in viral replication and NCCR rearrangement (Marshall et al. 2012). However, further studies are needed to understand what are the cellular pathways involved in the expression of the hematopoietic transcription factor Spi-B and how these pathways are regulated by the production of specific cytokines involved in the control of viral infection (Bellizzi et al. 2013).

Finally, the detection of the 217 G to T nucleotide transition in box F at the level of the binding site for the cellular transcription factor NF-1 is in agreement with literature data, which reported this point mutation in the European JCV strains (Agostini et al. 1996). However, in 4 urine samples collected respectively at t0, t1, t2, and t3 from 1 RRMS patient with a positive STRATIFY JCV^®^ at t3, a sequence of NCCR, characterized by a duplication of the binding site for the cellular transcription factor NF-1 in box F, was found without the 217 G to A nucleotide transition in the same box. It was noted that NF-1 increased the expression of early and late genes of JCV in glial cells, permissive to viral replication (Monaco et al. 2001; Shinohara et al. 1997). Moreover, the tandem repeated NF-1 binding sites on JCV NCCR could be considered determinants of specificity for glial cells and therefore determinants of neurotropism. Therefore, the duplication of the binding site for the cellular transcription factor NF-1 in box F, observed in NCCR isolated from urine samples, suggests that the natalizumab-induced alterations of immunosurveillance could determine a selective pressure on JCV variants with determinants of neurotropism and increased neurovirulence.

Sequence analysis of the viral VP1 was performed on samples of urine and plasma in order to determine the genotypes of JCV circulating in patients treated with natalizumab. The prevalence of genotypes 1A and 1B, most commonly found in European populations (Agostini et al. 2001), was observed. In the two patients with RRMS who, after 1 year of treatment with natalizumab had rearranged sequences in PBMC of the NCCR, were found genotype 1A and 1B, respectively.

In conclusion, although it has not been possible to identify a real correlation between the presence of variants neurovirulent and treatment with natalizumab, this study showed that during the spread of the virus into the host, you go to select particular sequences of the NCCR of JCV and that treatment with monoclonal antibody seems to have a role in the selection of these variants. However, it remains unclear what the viral and host factors are behind this selection process.

## Conclusion

In conclusion, for a more accurate stratification of the risk of PML in patients treated with natalizumab, the JC viruria assay would seem to be useful in identifying those patients with JCV-specific humoral response that is not yet detectable. In addition, the results of this study show the importance of the analysis of rearrangements of the NCCR of JCV. Currently, we do not know the significance of our finding regarding JCV NCCR analysis and, therefore, further investigation is warranted Moreover, the study of the molecular mechanisms involved in cell latency and reactivation of JCV in peripheral blood cells is of substantial importance in order to identify the missing pieces in the puzzle of the pathogenesis of PML. It is therefore essential to focus attention on cellular pathways, finely regulated by the host’s immune system, leading to reactivation of the virus under conditions of immunosuppression, since the treatment of PML with anti-viral drugs has proved to be ineffective.
